# Air Pollution and Watershed Research in the Central Sierra Nevada of California: Nitrogen and Ozone

**DOI:** 10.1100/tsw.2007.82

**Published:** 2007-03-21

**Authors:** Carolyn Hunsaker, Andrzej Bytnerowicz, Jessica Auman, Ricardo Cisneros

**Affiliations:** ^1^ USDA Forest Service, Pacific Southwest Research Station, Sierra Nevada Research Center, 2081 E. Sierra Avenue, Fresno, CA 93710, USA; ^2^ USDA Forest Service, Pacific Southwest Research Station, Forest Fire Laboratory, 4955 Canyon Crest Drive, Riverside, CA 92507, USA; ^3^ USDA Forest Service, Pacific Southwest Region, 1600 Tollhouse Rd., Clovis, CA 93611, USA

**Keywords:** air quality, water quality, Sierra Nevada, ozone, nitrogen

## Abstract

Maintaining healthy forests is the major objective for the Forest Service scientists and managers working for the U.S. Department of Agriculture. Air pollution, specifically ozone (O_3_) and nitrogenous (N) air pollutants, may severely affect the health of forest ecosystems in the western U.S. Thus, the monitoring of air pollution concentration and deposition levels, as well as studies focused on understanding effects mechanisms, are essential for evaluation of risks associated with their presence. Such information is essential for development of proper management strategies for maintaining clean air, clean water, and healthy ecosystems on land managed by the Forest Service. We report on two years of research in the central Sierra Nevada of California, a semi-arid forest at elevations of 1100–2700 m. Information on O_3_ and N air pollutants is obtained from a network of 18 passive samplers. We relate the atmospheric N concentration to N concentrations in streams, shallow soil water, and bulk deposition collectors within the Kings River Experimental Watershed. This watershed also contains an intensive site that is part of a recent Forest Service effort to calculate critical loads for N, sulfur, and acidity to forest ecosystems. The passive sampler design allows for extensive spatial measurements while the watershed experiment provides intensive spatial data for future analysis of ecosystem processes.

## INTRODUCTION

Within the U.S., the San Joaquin Valley in California had the worst ozone ($O_3$) air pollution from 1999–2004. Such air pollution rankings are based on human health standards, but ecosystem components can show negative effects or stress at pollutant levels lower than those set to protect humans. Air pollution can affect all portions of a forest ecosystem — vegetation, soil, soil water, and stream water. The Kings River

Project is located in the Sierra Nevada Mountains northeast of Fresno, California. The semi-arid forest of the southern Sierra Nevada receives air pollution mostly from the San Joaquin Valley. Here a large portion of nitrogen (N) is deposited as ammonia or nitric acid in the dry summer season. These pollutants are generated by mobile sources (trains, automobiles, and trucks), and agricultural and industrial activities in California's Central Valley and to some degree the San Francisco Bay Area. Some air pollution is also generated in the mountains by wildfires and by land managers using prescribed fire. Fire emissions are episodic, while the other types of emissions listed are chronic. Currently, regulatory air pollution agencies place most of their monitoring efforts in urban areas and focus on human health. Fire is a natural process in California's Mediterranean forests; however, the regulatory measures to protect human health result in very restrictive situations regarding the use of low-intensity fire to restore forest ecosystems. The Kings River Project is designed to address effects of air pollution, ozone ($O_3$), and N compounds, as well as land management activities on forest ecosystems in an integrated manner for a long-term period. Chemical concentrations in air, precipitation, soil water, and streams are being monitored to evaluate exposure and better understand ecosystem processes.

## STUDY AREA AND METHODS

The Kings River Experimental Watershed (KREW, www.fs.fed.us/psw/programs/snrc/water) is a long-term, intensive watershed experiment located within the larger Kings River Project (60,000 ha), which is a sustainable forest ecosystem study where management, research, and private industry are collaborating. The research area is located on the western side of the southern Sierra Nevada mountain range in a Mediterranean climate. The elevation ranges from 1100–2700 m for the air quality monitoring across the Kings River Project; the intensive N monitoring occurs on KREW, which is located in the mixed conifer forest, on granite-derived soils at the rain-snow transition zone. While chemistry data are being collected in many environmental media at KREW, this paper presents a brief overview and results for a small selection of the data being collected on air pollution and N cycle components in this area. The Providence Site of KREW consists of four headwater watersheds with perennial streams (P301, P303, P304, and D102); data collection began in water year (WY) 2002 (1 October 2001 through 30 September 2002).

The extensive air pollution monitoring across the Kings River Project was conducted using passive samplers[1] at 18 locations during two-week exposure periods in the 2003–2005 summer seasons (June–October). A general topographic map of the study area with location of the passive sampler monitoring sites shows steep elevation gradients and river valleys (Fig. 1). In this paper, integrated summer season average concentrations, as well as two-week concentrations during the highest period of pollution, the second half of July, are presented. The Kings River Project air pollution monitoring is now part of a larger study (38 locations) that includes the national parks to the north and south; this new study is addressing pollutant levels and pollutant transport from wildland fires in California.

KREW has objectives of characterizing watershed processes, evaluating effects from forest management (thinning and prescribed fire), and applying process models to better understand these forest ecosystems. Within the P301 watershed, an intensive (Level II) plot (Fig. 1) has been established consistent with the International Cooperative Programme on Assessment and Monitoring of Air Pollution Effects on Forests (ICP Forests). The Forest Service is evaluating the local forest condition, and the requirements and costs of these intensive monitoring plots. Nitrogenous compounds are measured every two weeks from bulk deposition collectors located under the forest canopy and in openings. These bulk collectors have a 10-cm diameter opening and are located 35 cm above the ground. Shallow soil water is also collected every two weeks using Prenart™ continuous vacuum lysimeters positioned at 13 and 26 cm in the mineral soil. The Prenart sampler is collocated with the throughfall bulk deposition samplers. The Kings River Project cluster of research activities provides both an extensive and intensive characterization of air pollution exposure and effects on forest ecosystem components, and research activities are expected to continue to expand.

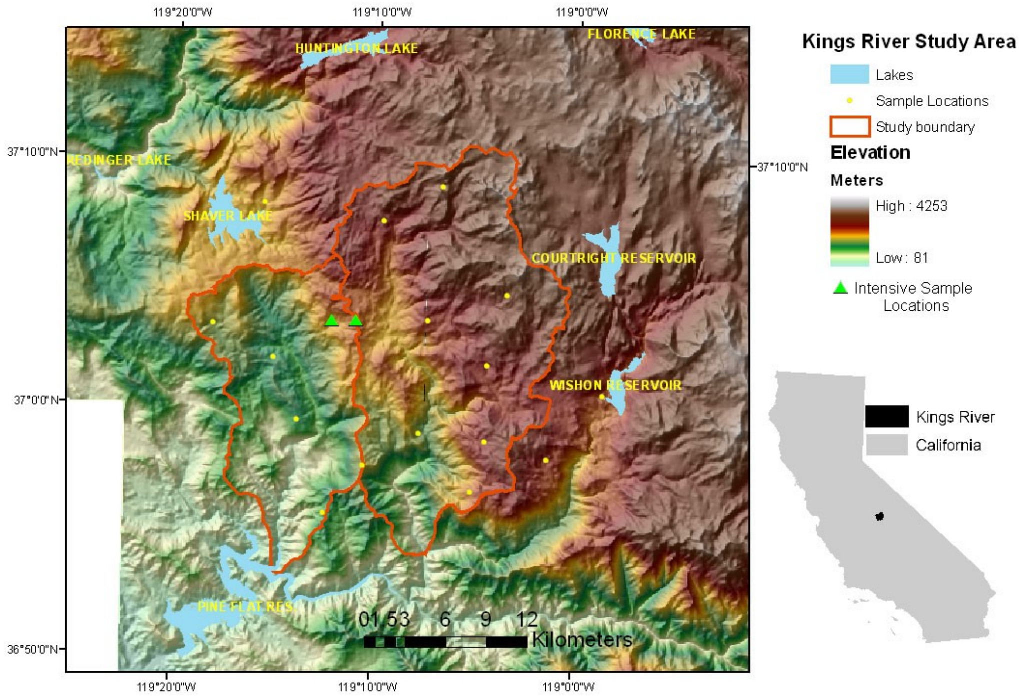

**FIGURE 1**. Locations of 18 passive samplers that provide extensive air pollution monitoring for the Kings River Project, Sierra National Forest, California. Two of these passive samplers are collocated with intensive ecosystem monitoring at the Providence Site of the Kings River Experimental Watershed

## RESULTS

### Air Pollution Concentrations

Ozone, nitric acid, and ammonia concentration descriptive statistics (range, mean, and standard deviation) were calculated for all 18 passive sampler sites (Table 1). Seasonal mean $O_3$ concentrations in a large portion of the study area were consistently higher than today's typical background of 50–60 ppb in the Northern Hemisphere mid-latitudes where atmospheric emission of $O_3$ precursors are high[2]. All but two sites exceeded 60 ppb as 2-weeklong averages sometime during the summer. Ozone distribution followed a pattern of being highest in the southwestern portion of the network and the lowest in the high elevation sites west of the Courtright and Wishon Reservoirs (Fig. 2A). This distribution correlates well with the summertime prevailing southwest winds bringing polluted air from California's Central Valley. In the second half of July, typical summer distribution of the pollutant was very high with concentrations approaching 115 ppb (Fig. 2B). The highest $O_3$ values were at elevations below 1200 m, and a general trend of diminishing $O_3$ concentrations with increasing elevation and distance from the pollution source area was seen (Table 1). Such a trend is consistent with other mountain studies in California[3] and Central Europe[4]. Various studies have also shown that $O_3$ concentrations at high elevation sites downwind from the pollution source areas may stay at high and potentially harmful levels for people and vegetation. This is because at night, no scavenging of $O_3$ by nitric oxide (NO), a phenomenon typical for urban areas, takes place as the polluted air masses move away from NO sources[5,6,7,8].

The highest concentrations of nitric acid ($HNO_3$) also occurred at the low elevation southwestern monitoring sites, and the lowest occurred at the high elevation sites west of the Courtright and Wishon Reservoirs (Fig. 2C). In addition, similar to $O_3$, a trend of elevated levels of $HNO_3$ in the eastern part of the study area was seen throughout the entire summer season (Fig. 2C) and was present in the second half

**TABLE 1**
**Two-Week Average $O_3$, $HNO_3$, and Ammonia ($NH_3$) Concentrations in the Kings River Project during the Summer of 2004**

| Location | Elevation (m) | $O_3$ (ppb) | | $HNO_3$ (μg/m$^3$) | | $NH_3$ (μg/m$^3$) | |
|---|---|---|---|---|---|---|---|
| | | Range | Mean (S.D.) | Range | Mean (S.D.) | Range | Mean (S.D.) |
| Forked Meadow | 1963 | 49–70 | 62(7) | 1.08–5.12 | 2.39 (1.66) | 2.92–6.81 | 4.32 (1.39) |
| Lower Providence Met | 1743 | 43–63 | 56 (7) | 1.06–4.75 | 2.08 (1.36) | 2.70–5.71 | 3.63 (1.12) |
| 9s09-9s10 Intersection | 2286 | 49–66 | 61 (7) | 1.26–3.01 | 2.04 (0.71) | 2.23–4.53 | 3.11 (0.82) |
| Dinkey Creek Wilderness | 2639 | 43–64 | 56 (8) | 0.98–5.09 | 2.21 (1.65) | 1.71–3.60 | 2.48 (0.82) |
| Cabin Meadow | 2158 | 44–65 | 57 (8) | 1.13–2.64 | 1.80 (0.78) | 2.19–4.87 | 3.45 (1.21) |
| Shaver Lake | 1713 | 44–63 | 53 (8) | 1.03–1.66 | 1.39 (0.32) | 1.71–4.64 | 3.76 (1.17) |
| Wishon Reservoir | 2042 | 56–68 | 62 (5) | 1.16–2.49 | 1.67 (0.52) | 2.11–5.52 | 3.91 (1.48) |
| Teakettle Exp. Forest | 1621 | 60–70 | 64 (4) | 1.11–3.40 | 2.32 (1.12) | 2.51–5.94 | 4.34 (1.37) |
| Lower Bull Met | 2219 | 46–58 | 52 (5) | 0.97–1.78 | 1.25 (0.37) | 2.50–6.99 | 4.15 (1.69) |
| Cow Heaven | 1890 | 55–72 | 65 (7) | 1.25–2.72 | 2.05 (0.68) | 2.53–6.37 | 4.75 (1.40) |
| Ross Crossing | 1353 | 50–65 | 57 (5) | 1.46–2.23 | 1.80 (0.35) | 3.42–10.99 | 5.29 (3.21) |
| Snow Corral | 2146 | 42–50 | 46 (3) | 0.81–2.08 | 1.45 (0.54) | 2.37–3.94 | 2.97 (0.70) |
| Bear Creek | 2700 | 50–62 | 57 (5) | 1.10–4.34 | 1.93 (1.37) | 1.23–4.04 | 2.94 (0.94) |
| Blue Canyon Helipad | 1219 | 58–115 | 74 (23) | 1.88–5.05 | 3.35 (1.26) | 3.71–5.32 | 4.58 (0.64) |
| Fence Meadow | 1600 | 59–78 | 70 (7) | 2.55–3.61 | 3.00 (0.52) | 4.85–12.50 | 4.85 (3.34) |
| Granite Slab | 1469 | 56–79 | 67 (9) | 2.47–7.34 | 4.27 (1.65) | 4.40–7.12 | 5.79 (0.94) |
| Barnes Mountain | 1122 | 63–77 | 71 (5) | 2.62–6.30 | 3.88 (1.27) | 4.39–6.45 | 5.34 (0.75) |
| Pine Flat Vista | 1122 | 62–80 | 70 (7) | 2.83–8.54 | 4.88 (2.02) | 4.87–7.31 | 6.08 (1.03) |

of July (Fig. 2D). During the summer season, the $HNO_3$ concentrations at KREW ranged from 0.81–8.54 μg/m$^3$ (Table 1), values similar to the low and moderately polluted regions of the San Bernardino Mountains near the Los Angeles Basin (5–10 μg/m$^3$)[9]. Monitoring results for $O_3$ and $HNO_3$ also indicate that polluted air masses from the San Joaquin Valley could move deeply into the Sierra Nevada range in a northeast direction along the Kings River Valley (see Fig. 1).

Similar to $O_3$ and $HNO_3$, the highest concentrations of ammonia ($NH_3$) occurred at the low-elevation southwestern monitoring sites to the north and east of Pine Flat Reservoir, while the lowest levels were recorded at the northeastern high-elevation sites (Fig. 2E). In the second half of July (Fig. 2F), very high levels at the Fence Meadow site in the southern part of the study area were recorded (see Fig. 1). Eight sites had some $NH_3$ values above 6 μg/m$^3$ sometime during the summer with one location (Pine Flat Vista) having a mean value for the entire season above 6 μg/m$^3$ (Table 1). Ammonia concentrations were higher than those reported in Barton Flats of the San Bernardino Mountains[10] and in Ash Mountain, Sequoia National Park[6]. For a comparison, the maximum two-week mean $NH_3$ concentration at Ash Mountain was 8 μg/m$^3$, whereas two of the sites in the Kings River Study reached concentrations of 12.50 (Fence Meadow) and 11 μg/m$^3$ (Ross Crossing).

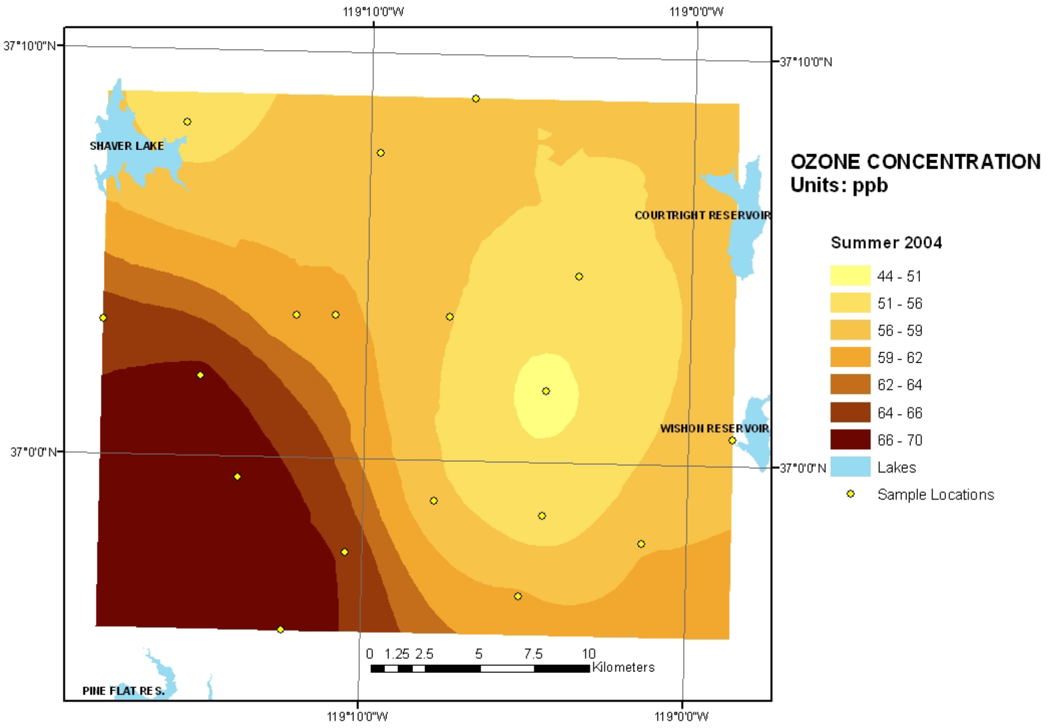

A

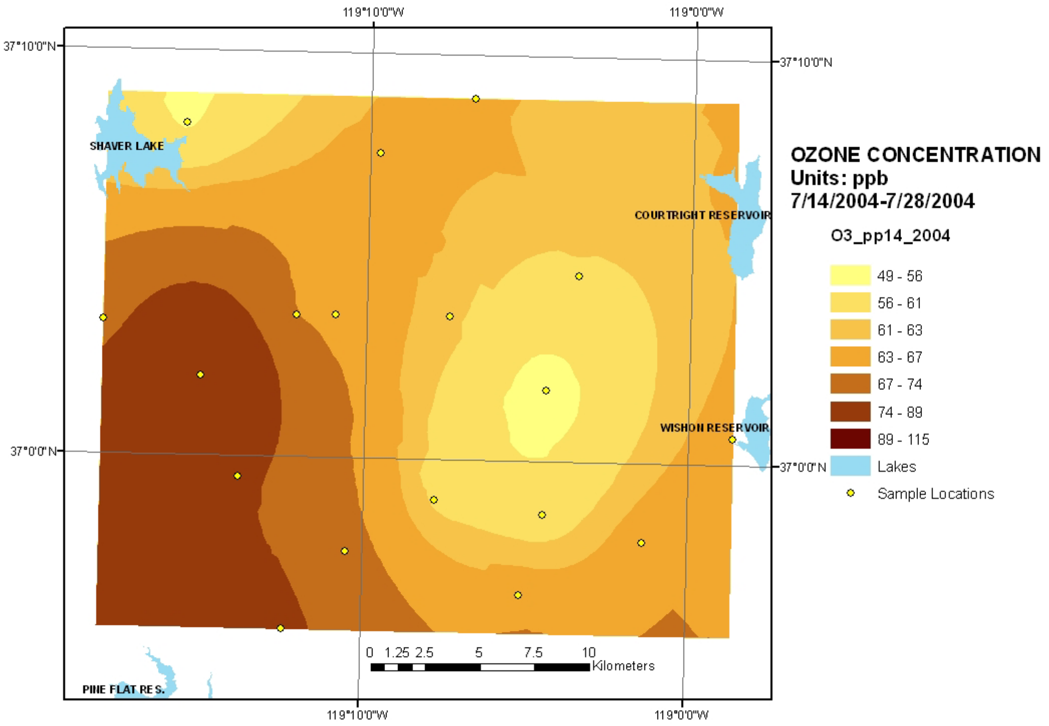

B

**FIGURE 2**. (A) Summer season mean $O_3$ concentrations at the Kings River Project during 2004. (B) Two-week mean $O_3$ concentrations in the second half of July. (C) Summer season mean $HNO_3$ concentrations at the Kings River Project during 2004. (D) Two-week mean $HNO_3$ concentrations in the second half of July. (E) Summer season mean $NH_3$ concentrations at the Kings River Project during 2004. (F) Two-week mean $NH_3$ concentrations in the second half of July.

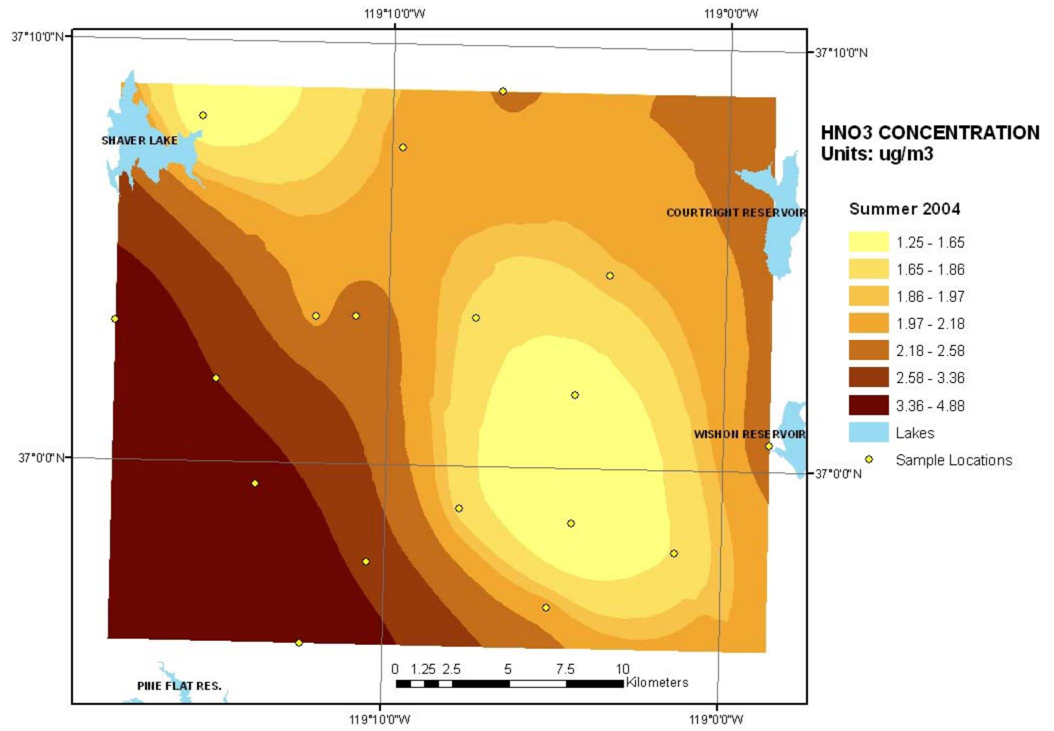

**FIGURE 2C**

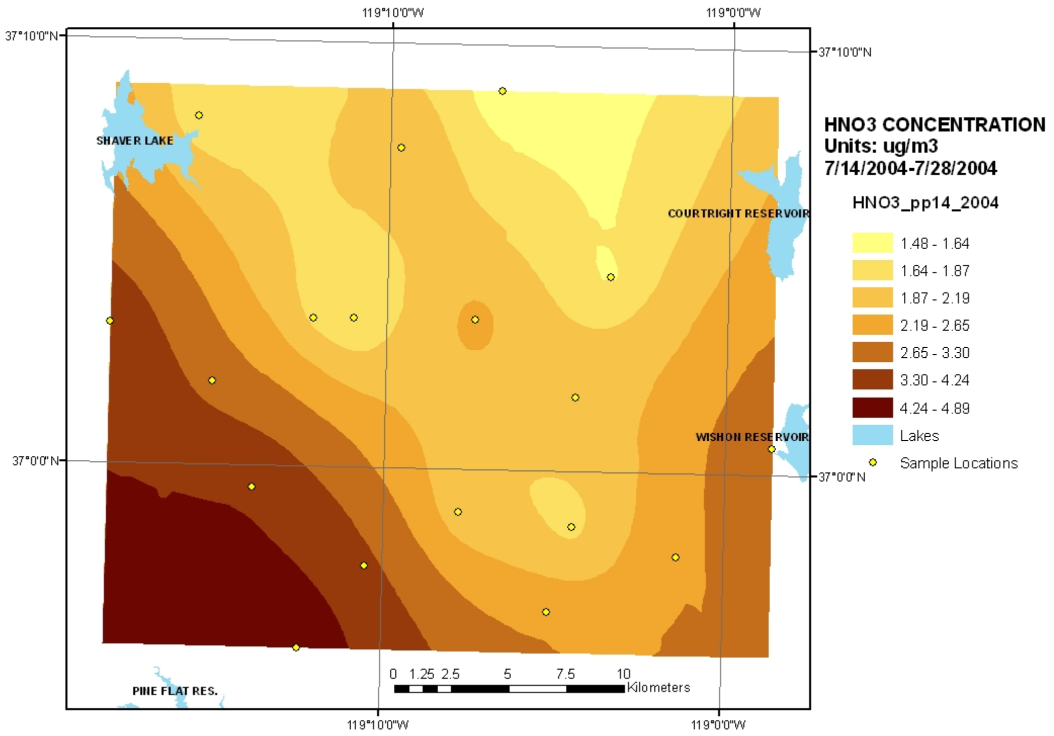

**FIGURE 2D**

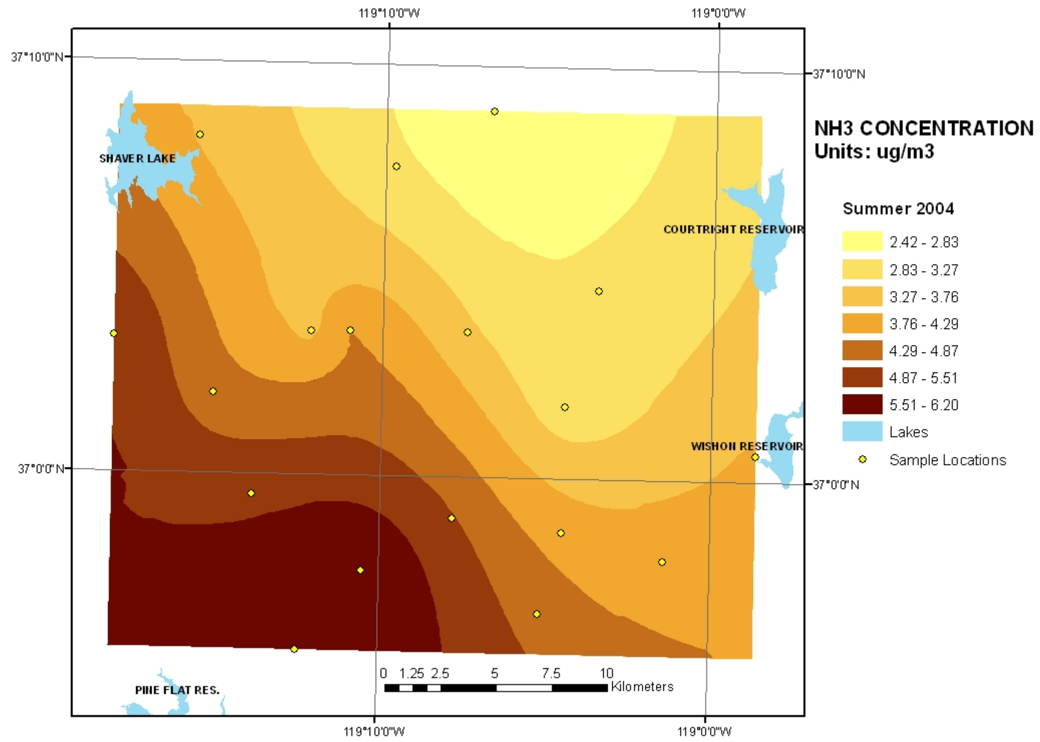

**FIGURE 2E**

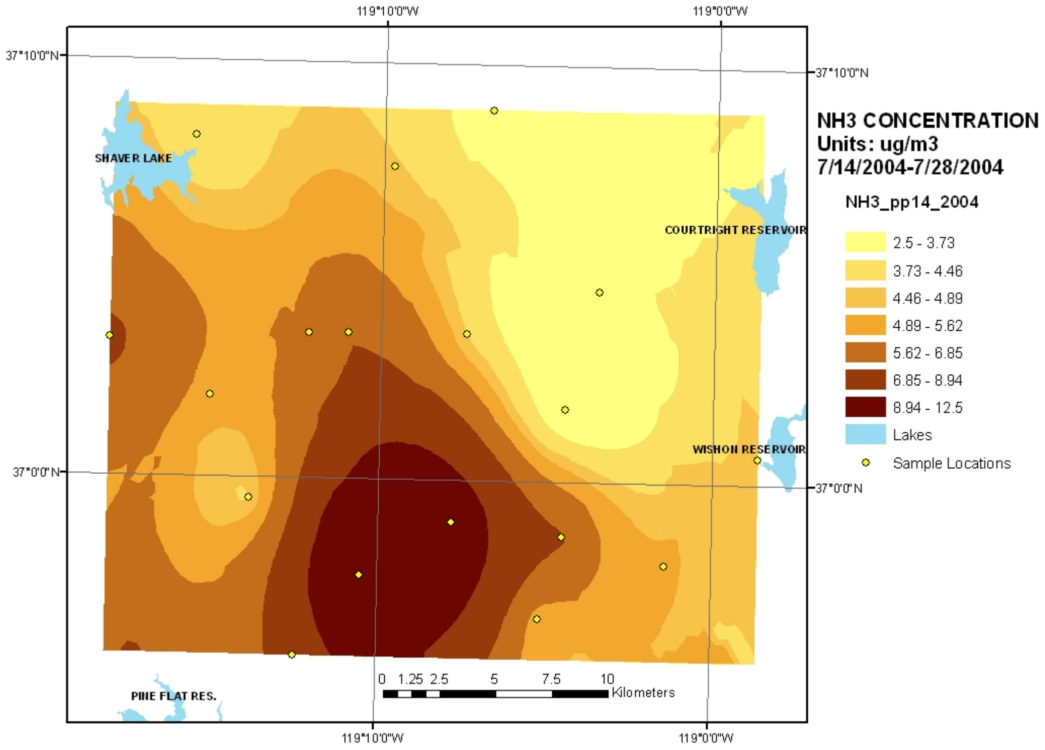

**FIGURE 2F**

Steep gradients of $NH_3$ and $HNO_3$, much more pronounced than for $O_3$, were observed in this study and may be explained by the high deposition velocities and chemical reactivity of these two N pollutants[11]. On the other hand, ambient $O_3$ may be transported over long distances, thousands of kilometers away from the source areas[12]. The high levels of $O_3$ and $HNO_3$ in the second half of July could have been caused by a prescribed fire that was occurring to the east of the project area, in the Sequoia National Park, during July and August. Background levels of $HNO_3$ in summer (e.g., Eastern Brook Lake, eastern Sierra Nevada) should not exceed 0.4 $\mu g/m^3$[13], while those for $NH_3$ in remote mountain locations in North America should be less than 1 $\mu g/m^3$[14]. The results confirm that in the western Sierra Nevada, levels of $NH_3$ are higher than those of $HNO_3$, mainly due to agricultural emissions in the San Joaquin Valley[6]. During the polluted summer season, both pollutants are the main drivers of elevated N deposition because of their high concentrations and deposition velocities[14].

## Nitrogen Deposition

Bulk deposition collectors (also called snowmelt collectors) are located under the forest canopy to measure throughfall (six collectors) and in a nearby clearing (one collector) on each watershed; water is collected every two weeks during the wet season. The wet season is usually November through May. Nitrate and ammonium concentrations for WY2003 and 2004 are presented for watershed P303 at the intensive sampling area (Fig. 3). Values are usually highest in March and April. Median nitrate concentrations were higher in 2004, i.e., wider range of values (0.75–2.5 mg $NO_3$/l) than in 2003 (0.1–0.9 mg $NO_3$/l). However, 2003 had more values above 0.1 mg/l. Ammonium concentrations were similar during the 2 years. Wet season throughfall deposition of N ($NO_3$-N + $NH_4$-N) ranged from 1.1–1.7 kg/ha/year and 0.5–1.1 kg/ha/year among the four watersheds in 2003 and 2004, respectively. Open bulk deposition fluxes were similar to the throughfall values. In addition to these bulk deposition collectors, KREW has several measurement devices that will contribute to the calculation of N deposition to the forest: ground-level resin lysimeters, above-ground resin columns, and the passive samplers discussed previously. In 2004, watershed P303 had atmospheric concentrations in the middle of the range measured for $HNO_3$ (~2 $\mu g/m^3$) and $NH_3$ (~4 $\mu g/m^3$). Nitrogen loadings of 7–12 kg/ha/year[14,15] have been reported for mountain areas immediately north and south of the Kings River Project.

## Nitrogen in Soil Water and Streams

Most N samples from stream water and shallow soil water are below detection level (0.05 mg/l); however, we present all measured values for the soil water samples in WY2003 and 2004 (Fig. 4). In WY2004, nitrate concentrations above 0.05 mg/l were never measured at either 13 or 26 cm of mineral soil; only a few sampling periods for an occasional watershed had ammonium concentrations above 0.05 mg/l. In WY2003, both nitrate and ammonium were measured above 0.05 mg/l more often than in 2004, especially at the 13-cm depth. From February to May, ammonium concentrations ranged from 0.05–0.14 mg/l, and nitrate ranged from 0.05–0.5 mg/l. These are very low concentrations of N as would be expected for a relatively undisturbed forest watershed. There does not appear to be a substantial difference in concentrations between the two depths. Since the Providence Site is at the rain-snow transition, it experiences a lot of variability in the amount of snow pack, and the melting and freezing process. Higher N concentrations in soil water during spring is likely driven by the snowmelt process. Total N deposition and concentrations in shallow soil water were approximately 50% higher in WY2003 than WY2004.

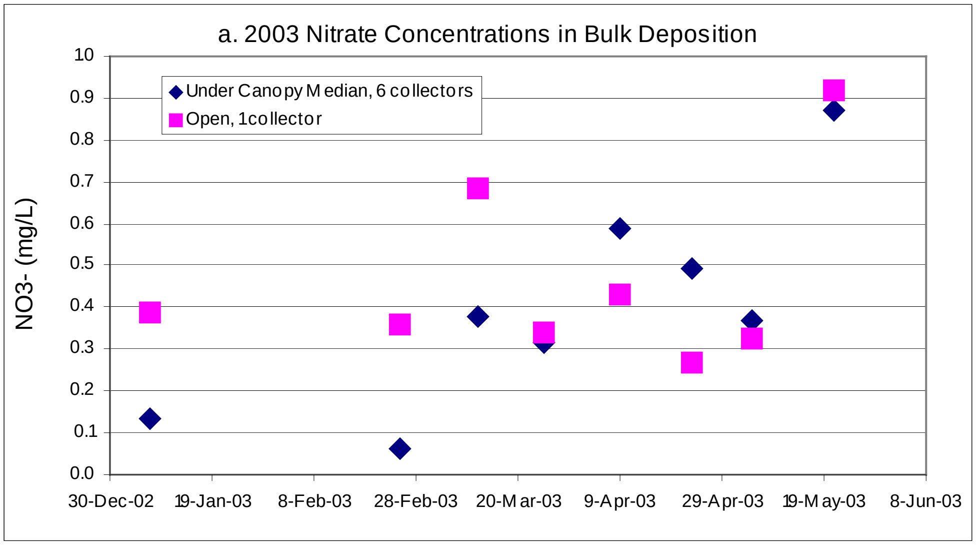

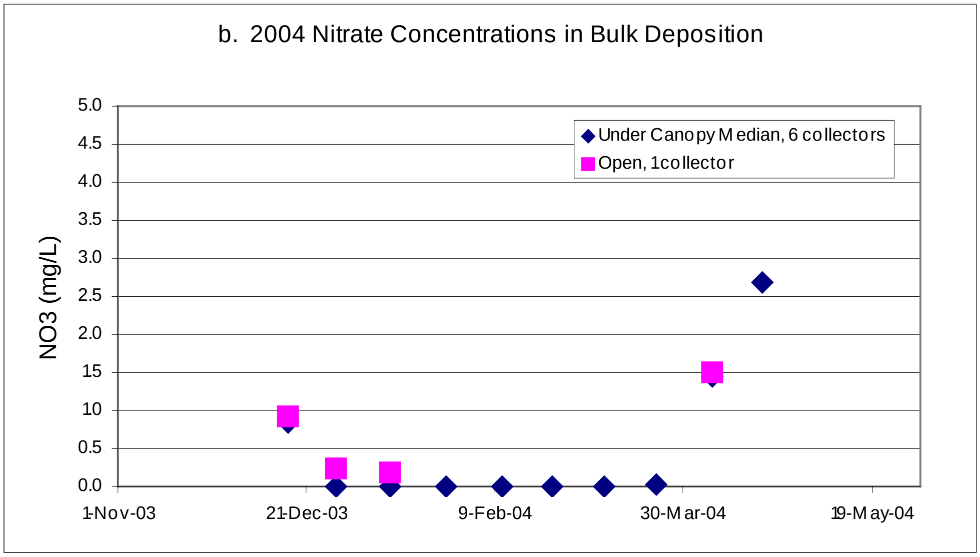

**FIGURE 3**. Bulk deposition (snowmelt) samplers, open and throughfall, at watershed 303 in the Providence Site. Values reported are medians. (a) 2003 nitrate concentrations; (b) 2004 nitrate concentrations; (c) 2003 ammonium concentrations; (d) 2004 ammonium concentrations.

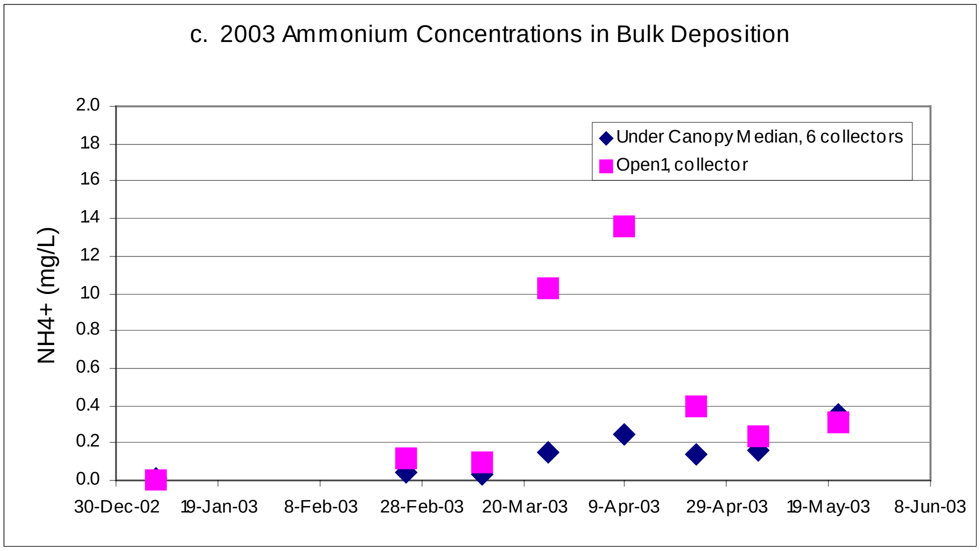

c. 2003 Ammonium Concentrations in Bulk Deposition
NH4+ (mg/L)
2.0
18
16
14
12
10
0.8
0.6
0.4
0.2
0.0
30-Dec-02
19-Jan-03
8-Feb-03
28-Feb-03
20-Mar-03
9-Apr-03
29-Apr-03
19-May-03
8-Jun-03
Under Canopy Median, 6 collectors

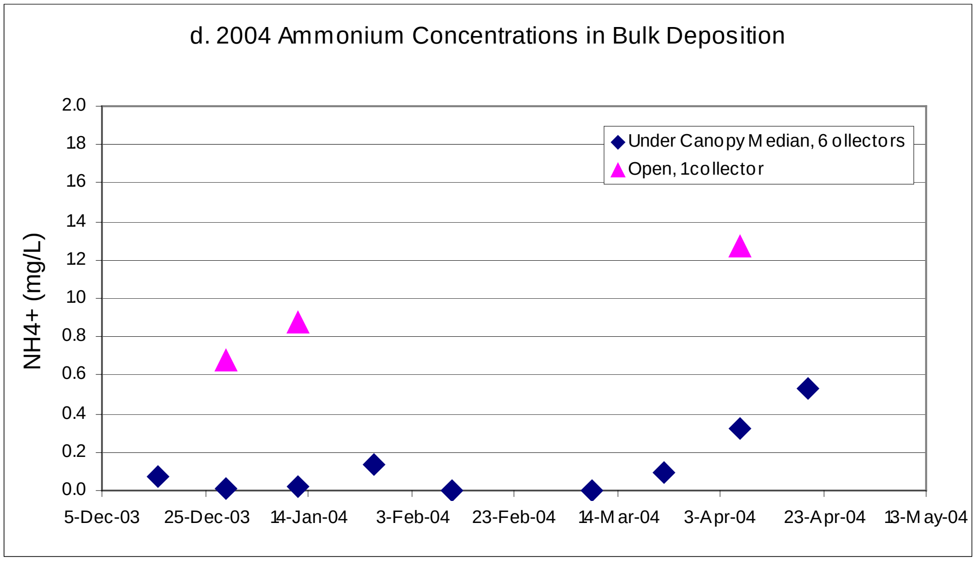

Open1, collector d. 2004 Ammonium Concentrations in Bulk Deposition
NH4+ (mg/L)
2.0
18
16
14
12
10
0.8
0.6
0.4
0.2
0.0
5-Dec-03
25-Dec-03
14-Jan-04
3-Feb-04
23-Feb-04
14-Mar-04
3-Apr-04
23-Apr-04
13-May-04
Under Canopy Median, 6 ollectors
Open, 1collector

**FIGURE 3 c-d**

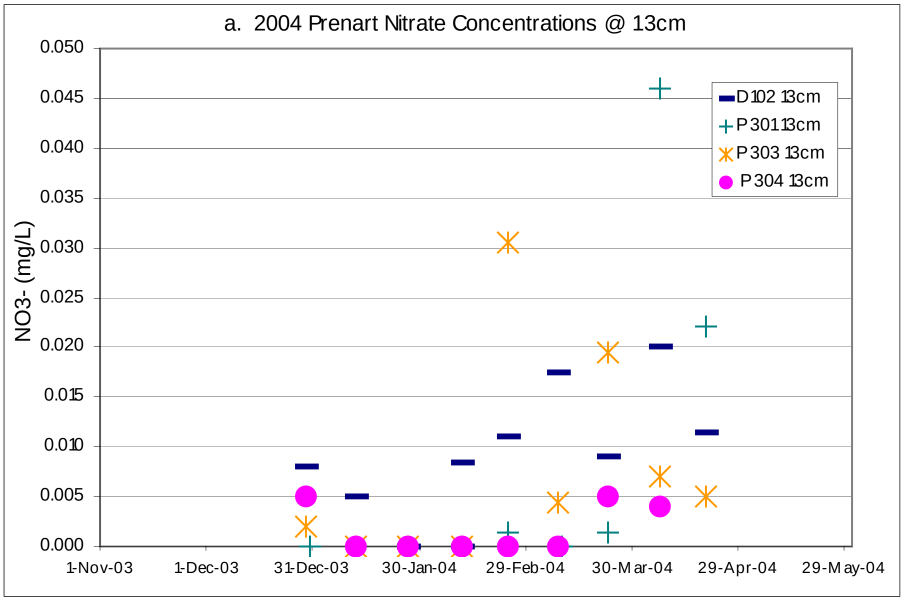

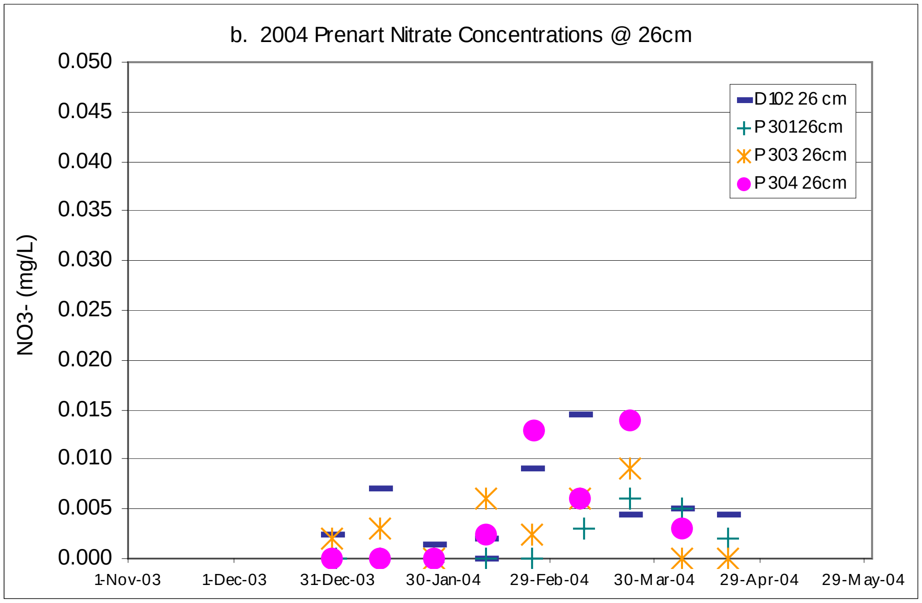

**FIGURE 4**. Continuous vacuum lysimeter sampling of soil water at the Providence Site indicates N concentrations in shallow soil water are often below 0.05 mg/l. Values reported are medians. (a) 2004 nitrate concentrations at 13-cm mineral soil depth; (b) 2004 nitrate concentrations at 26-cm depth; (c) 2004 ammonium concentrations at 13-cm depth; (d) 2004 ammonium concentrations at 26-cm depth; (e) 2003 ammonium concentrations at 13-cm depth; (f) 2003 ammonium concentrations at 26-cm depth; (g) 2003 nitrate concentrations at 13-cm depth; (h) 2003 nitrate concentrations at 26-cm depth.

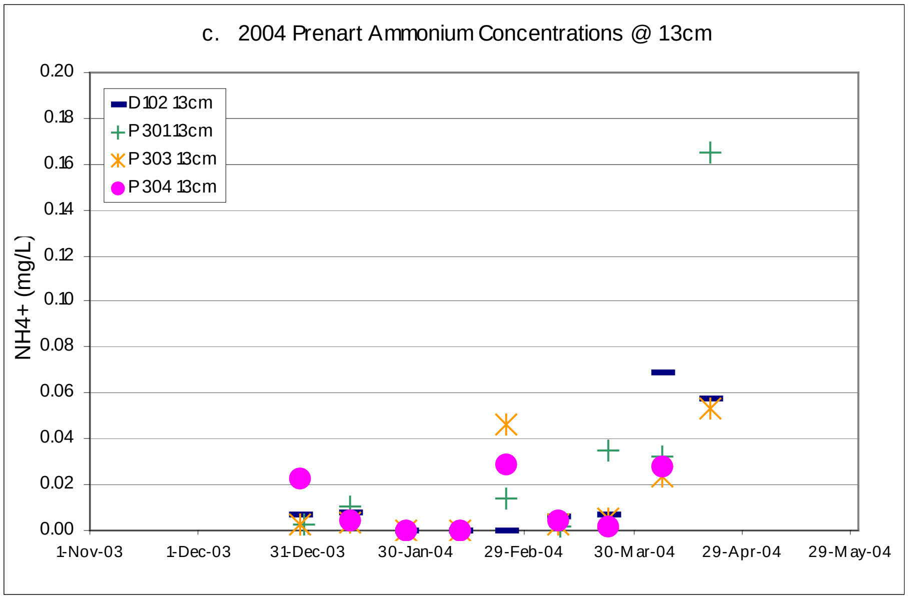

c. 2004 Prenart Ammonium Concentrations @ 13cm
NH4+ (mg/L)
0.20
0.18
0.16
0.14
0.12
0.10
0.08
0.06
0.04
0.02
0.00
D102 13cm
P301 13cm
P303 13cm
P304 13cm
1-Nov-03
1-Dec-03
31-Dec-03
30-Jan-04
29-Feb-04
30-Mar-04
29-Apr-04
29-May-04

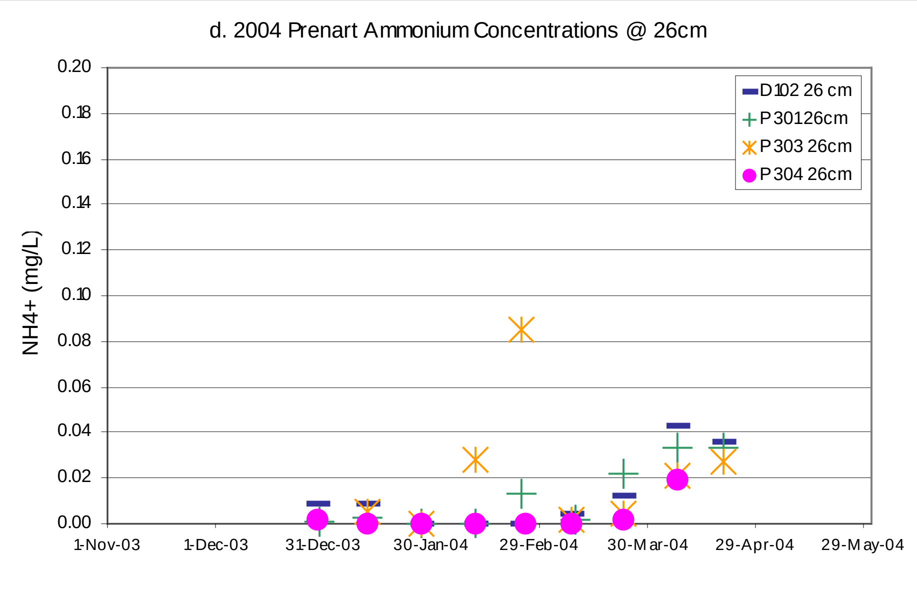

d. 2004 Prenart Ammonium Concentrations @ 26cm
NH4+ (mg/L)
0.20
0.18
0.16
0.14
0.12
0.10
0.08
0.06
0.04
0.02
0.00
D102 26 cm
P301 26cm
P303 26cm
P304 26cm
1-Nov-03
1-Dec-03
31-Dec-03
30-Jan-04
29-Feb-04
30-Mar-04
29-Apr-04
29-May-04

**FIGURE 4c-d**

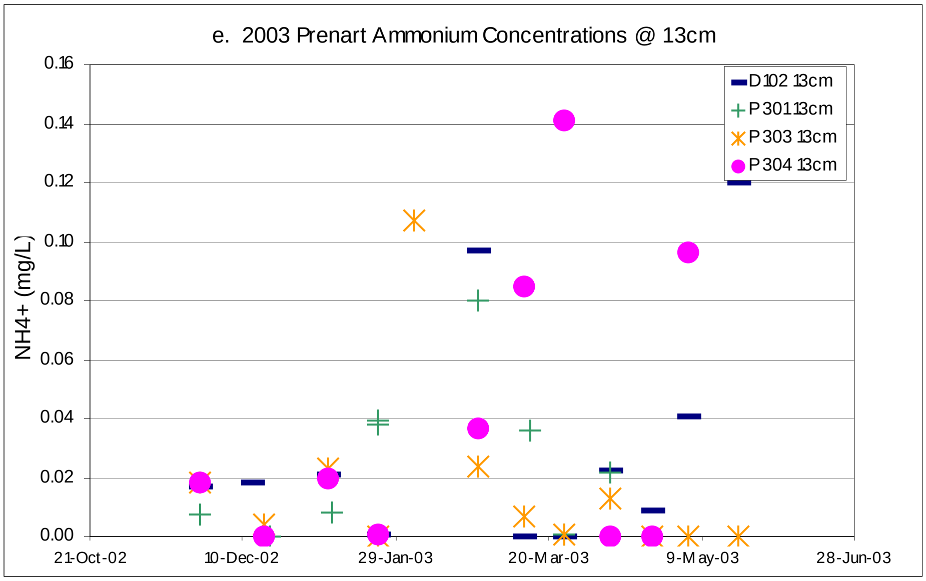

e. 2003 Prenart Ammonium Concentrations @ 13cm
NH4+ (mg/L)
0.16
0.14
0.12
0.10
0.08
0.06
0.04
0.02
0.00
21-Oct-02
10-Dec-02
29-Jan-03
20-Mar-03
9-May-03
28-Jun-03
D102 13cm
P301 13cm
P303 13cm

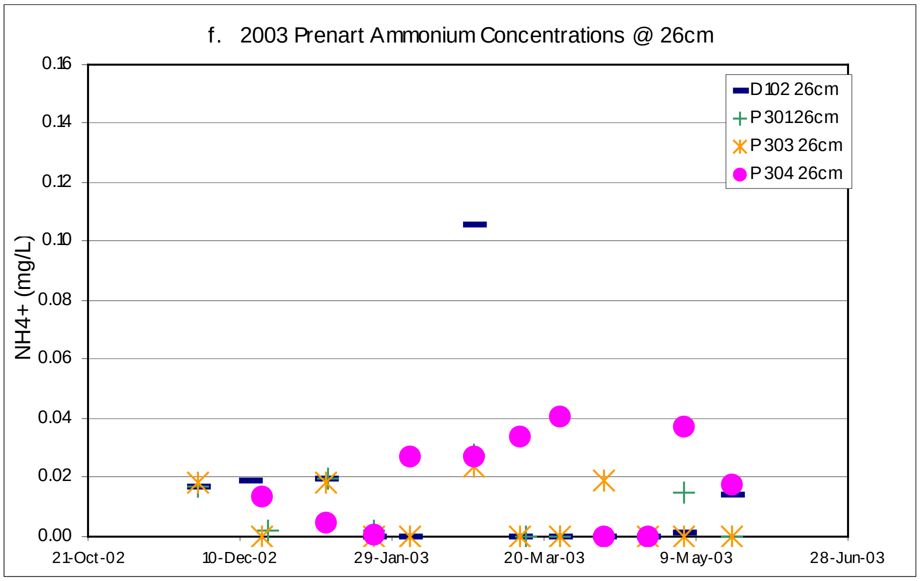

P304 13cm f. 2003 Prenart Ammonium Concentrations @ 26cm
NH4+ (mg/L)
0.16
0.14
0.12
0.10
0.08
0.06
0.04
0.02
0.00
21-Oct-02
10-Dec-02
29-Jan-03
20-Mar-03
9-May-03
28-Jun-03
D102 26cm
P301 26cm
P303 26cm
P304 26cm

**FIGURE 4 e-f**

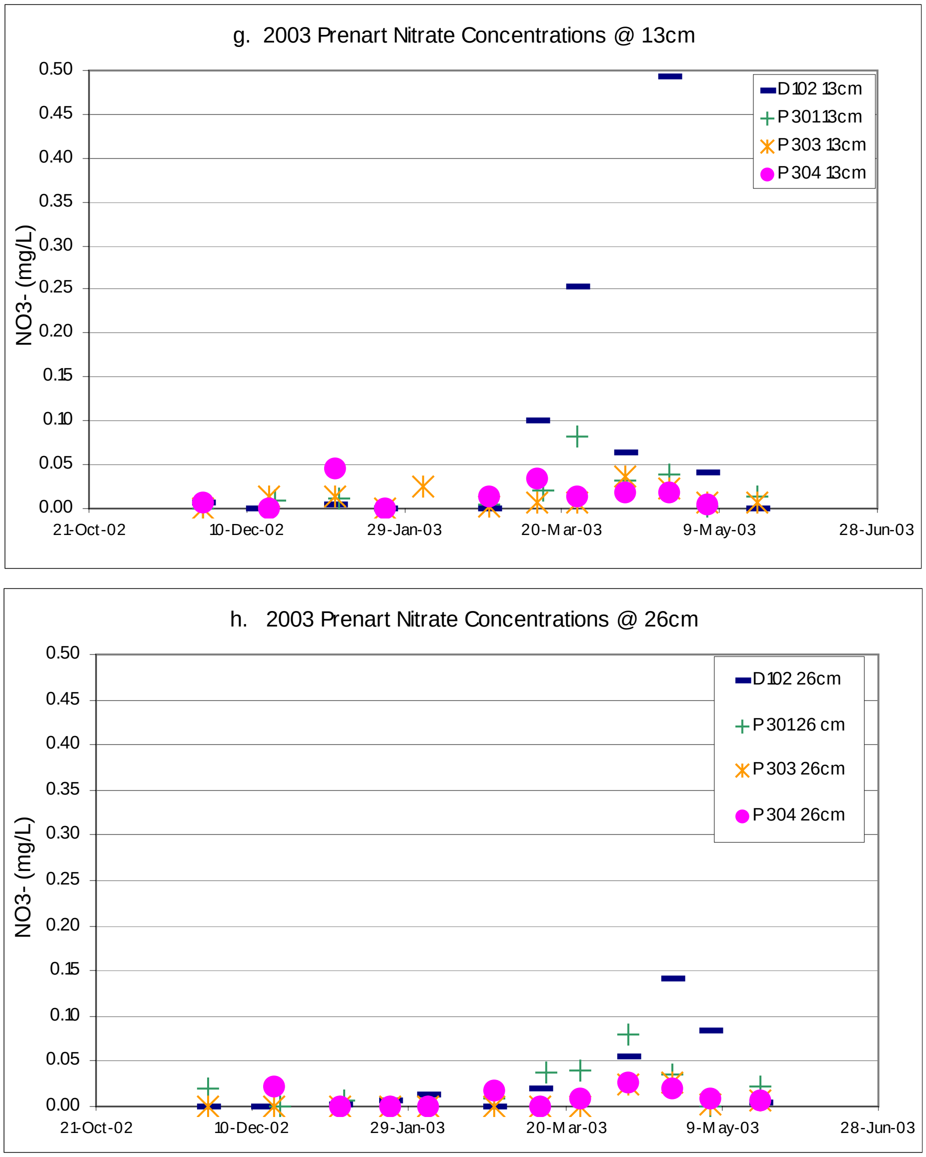

**FIGURE 4 g-h**

## SUMMARY AND CONCLUSIONS

Our 2004 air pollution data confirm that the Kings River Project area is receiving high exposures to $O_3$ and N compounds, and forests are likely experiencing stress from $O_3$ pollution. In future analyses, these data will be compared to measurements taken in 2003 and 2005 to confirm the patterns and concentrations. While atmospheric concentrations of N pollutants are moderately elevated, there is no evidence that significant amounts of N are moving through the soils into the streams. A consistent pattern of a slight increase in N is present during spring in soil water, especially in WY 2003, when deposition was twice as high as 2004; this is likely caused by snowmelt processes. Under current conditions, forest vegetation seems to be taking up most available N. To better understand ecosystem processes, the measurements described here, as well as others, will continue during and after mechanical thinning and prescribed fire treatments on these watersheds.

## FUTURE ANALYSES

Data are provided here from only a portion of the years that were monitored and the instruments being used at the Kings River Experimental Watershed. Four meteorological sites are maintained on KREW and, where possible, instruments and collectors are collocated within watersheds. Information on ambient concentrations of the individual N gaseous pollutants can be used for estimates of N dry deposition (fluxes). For such calculations, information on deposition velocity of individual gases and leaf area index of plant canopies is also needed. Above-ground resin columns, on-ground resin lysimeters at 150-m spacing, snowmelt bulk deposition collectors, and a new National Atmospheric Deposition Program site will provide data for future estimates of N deposition. Flux estimates are planned from the atmosphere to the ground, from the ground surface into shallow soil water, and from the ground or shallow soil water into the streams. Flux changes will be followed through tree thinning and prescribed fire treatments.

## ACKNOWLEDGMENTS

The authors thank the many Forest Service employees and students that collected data during these years. We also thank Dr. Dale Johnson, University of Nevada Reno, for his help in designing portions of KREW and Trent Procter, Pacific Southwest Region, for providing management support to this activity.
